# A Bacteriophage Cocktail Significantly Reduces *Listeria monocytogenes* without Deleterious Impact on the Commensal Gut Microbiota under Simulated Gastrointestinal Conditions

**DOI:** 10.3390/v14020190

**Published:** 2022-01-19

**Authors:** Rasmus Riemer Jakobsen, Jimmy T. Trinh, Louise Bomholtz, Signe Kristine Brok-Lauridsen, Alexander Sulakvelidze, Dennis Sandris Nielsen

**Affiliations:** 1 Section of Microbiology and Fermentation, Department of Food Science, Faculty of Science, University of Copenhagen, 1958 Frederiksberg, Denmark; s193261@student.dtu.dk (L.B.); signe.k.brok@sund.ku.dk (S.K.B.-L.); dn@food.ku.dk (D.S.N.); 2 Intralytix, Inc., 8681 Robert Fulton Drive, Columbia, MD 21046, USA; jimmyttri@gmail.com (J.T.T.); asulakvelidze@intralytix.com (A.S.)

**Keywords:** *Listeria monocytogenes*, phage therapy, gut microbiome

## Abstract

In this study, we examined the effect of a bacteriophage cocktail (tentatively designated as the Foodborne Outbreak Pill (FOP)) on the levels of *Listeria monocytogenes* in simulated small intestine, large intestine, and Caco-2 model systems. We found that FOP survival during simulated passage of the upper gastrointestinal was dependent on stomach pH, and that FOP robustly inhibited *L. monocytogenes* levels with effectiveness comparable to antibiotic treatment (ampicillin) under simulated ilium and colon conditions. The FOP did not inhibit the commensal bacteria, whereas ampicillin treatment led to dysbiosis-like conditions. The FOP was also more effective than an antibiotic in protecting Caco-2 cells from adhesion and invasion by *L. monocytogenes* (5-log reduction vs. 1-log reduction) while not triggering an inflammatory response. Our data suggested that the FOP may provide a robust protection against *L. monocytogenes* should the bacterium enter the human gastrointestinal tract (e.g., by consumption of contaminated food), without deleterious impact on the commensal bacteria.

## 1. Introduction

*Listeria monocytogenes* is a facultative anaerobic Gram-positive bacterium responsible for many cases of foodborne illness, manifesting as gastroenteritis, meningitis, encephalitis, mother-to-fetus infections, and septicemia. Although the annual number of *L. monocytogenes* infections globally is moderate, with 2502 confirmed cases in the EU in 2017 [1] and an estimated 23,150 global cases in 2010, the mortality rate of infected individuals is considerable at 20–30% [2]. The diverse clinical manifestations of *L. monocytogenes* are a result of its ability to enter both macrophages and other cell types, where it can survive and multiply [3]. Crossing the epithelial barrier by adhering to and invading intestinal epithelial cells gives access to internal organs, the first step toward systemic infection of the host.

Bacteriophages (or “phages” for short) are viruses that attack bacteria in a host-specific manner, acting as self-replicating antimicrobials. Lytic phages replicate through the lytic cycle, in which the phage infects the bacterial cell, uses the bacterial replication and translation machinery to replicate, and then lyses the bacteria to release new phage particles. Compared to antibiotics, phages are: (i) host specific, often only targeting specific strains within a specific species or (more seldom) within a limited number of closely related species; and (ii) unable to infect and replicate in eukaryotic cells. These factors make phages a promising means of targeted bacterial eradication within a microbial population without collateral damage to commensal bacteria [4]. Additionally, the mechanisms by which antibiotics and phages kill bacteria are fundamentally different, meaning potential bacterial resistances arise differently [5]. Consequently, phages can kill multidrug-resistant bacteria that available antibiotics cannot, and the emergence of resistance to both would likely be mutually exclusive, allowing phage–antibiotic complementary treatments [6]. Phages may also be utilized for biocontrol applications; e.g., phages are added to food products to reduce contamination with foodborne bacteria and consequent risk of foodborne diseases [7]. In 2006, the United States Food and Drug Administration (FDA) approved ListShield™ (one of the components of the FOP preparation that specifically targets *Listeria monocytogenes*) by asserting that it complies with FDA food-additive regulations for direct application to meat and poultry products that meet the ready-to-eat definition (21 CFR § 172.785). This was the first phage-based food-safety preparation ever approved by the FDA. Another *Listeria monocytogenes*-targeting phage preparation, Listex™, was given generally recognized as safe (GRAS) status by the FDA shortly afterwards [8]. Several other phage preparations for food safety applications have since been approved by the FDA and are currently available for sale in the United States [9].

Preventive or therapeutic use of lytic phages is potentially an attractive approach for enhancing natural gut defenses against pathogenic bacteria such as *L. monocytogenes*, and/or as a complement to the current standard of care for various bacterial infections, including antibiotic treatment [10]. For optimal efficacy, orally administered phages must first pass through several harsh environments during GI passage, including low pH in the stomach and pancreatic enzymes, and bile salt in the small intestine. All these factors may reduce phage stability, destroying them or rendering them less active. Despite the long history of using phages therapeutically [11], the pharmacokinetics of orally administered phage preparations is still not fully delineated, and it could be highly variable between phages and strongly affected by individual variations in gastrointestinal tract conditions and microbiota composition [12]. Furthermore, there is striking paucity of data on the impact GI passage on *Listeria* phage viability and their ability to lyse their targeted bacteria in the GI tract after such passage. The *Listeria* phages investigated in this study were components of the Foodborne Outbreak Pill (FOP) (Intralytix, Inc., Columbia, MD, USA) phage cocktail. The FOP is a mixture of three different phage preparations containing lytic phages targeting *Listeria monocytogenes*, *Salmonella* spp., and Shiga toxin producing *Escherichia coli* (STEC) [13,14]. The goals of this study were to test: (i) survivability of *Listeria monocytogenes* phages under conditions mimicking those found in the stomach; (ii) the potential of using the FOP bacteriophage cocktail to selectively target *L. monocytogenes* in the gut, and assess its impact on representative commensal bacteria using simulated human GI conditions (small and large intestines); and (iii) the ability of the same phage cocktail to protect Caco-2 cells from adhesion and invasion by *L. monocytogenes.*

## 2. Materials and Methods

### 2.1. Bacteriophage Cocktail

The FOP bacteriophage cocktail was created by Intralytix Inc. by combining, in approximately equal concentrations, three FDA-cleared commercial phage preparations currently marketed in the United States for food-safety applications: ListShield™ (six phages active against *Listeria monocytogenes*), EcoShield PX™ (three phages active against STEC), and SalmoFresh™ (six phages active against *Salmonella enterica*). Therefore, the FOP cocktail contained 15 distinct lytic phages that together targeted *L. monocytogenes*, *Salmonella* spp., and STEC, including O157:H7 strains (Table S1). The FOP cocktail in liquid form was an aqueous solution (pH 6.5–7.5) that was clear to slightly milky in color, and was stored refrigerated (2–8 °C) in the dark until use. For the stock FOP solution used in the experiments, the number of viable bacteriophages against *L. monocytogenes* was determined to be 10.83 log PFU/mL by plaque assay [15] using *L. monocytogenes* strain LM114 on Luria–Bertani (LB)+ (10 g tryptone/L, 5 g yeast extract/L, 10 g NaCl/L, 0.02 M MgCl_2_, 0.001 M CaCl_2_; pH = 7.0) 1.5% agar.

### 2.2. Bacterial Strains

The *L. monocytogenes* strains, LM114 (serotype 4b) and LM396 (serotype 1/2a), were isolated from food-processing plants, and provided by Intralytix. These strains were confirmed to be susceptible to the FOP cocktail via plaque assay [15]. Both *L. monocytogenes* strains were propagated in LB broth (10 g tryptone, 5 g yeast extract, 10 g NaCl/L; pH = 7.0) at 37 °C with shaking (90 rpm). Quantification of all strains was performed via determination of colony-forming units on LB agar.

### 2.3. Small Intestinal Model System

#### 2.3.1. Small Intestine In Vitro Simulation

To simulate passage of phages through the human stomach and small intestine, we used a recently developed dynamic in vitro model (TSI) [16] using fed-state parameters (1 h stomach passage, pH 4, bile salts = 10 mM, pancreatic juice = 100 U/mL) [16]. For preliminary studies of phage viability during stomach passage, fasted-state parameters (30 min stomach passage at pH 2, bile salts = 4 mM, pancreatic juice = 40 U/mL) were tested for comparison. The TSI model consisted of five reactors with working volumes of 12 mL, each simulating the small intestine of one individual. The pH and temperature were maintained at physiologically relevant levels, while simulated intestinal media, food, bile salts, and digestive enzymes levels were established and maintained to simulate passage through stomach, duodenum, jejunum, and ileum as previously described [16].

#### 2.3.2. Consortium of Small Intestinal Bacteria

To simulate a normal, healthy, small intestine microbiome, a consortium of 7 bacterial species were selected to represent a healthy ileal microbiota [17,18] (Table 1). All bacteria were acquired from the German Collection of Microorganisms and Cell Cultures (Leibniz Institute DSMZ, Braunschweig, Germany) and prepared and enumerated as described previously [16]. A total of 1 mL of consortium containing 10^8^ CFU mL^−1^ small intestinal bacteria was added to each TSI reactor.

### 2.4. Bacteriophage Impact on L. monocytogenes during Stomach and Small Intestine Passage

**Figure 1:**
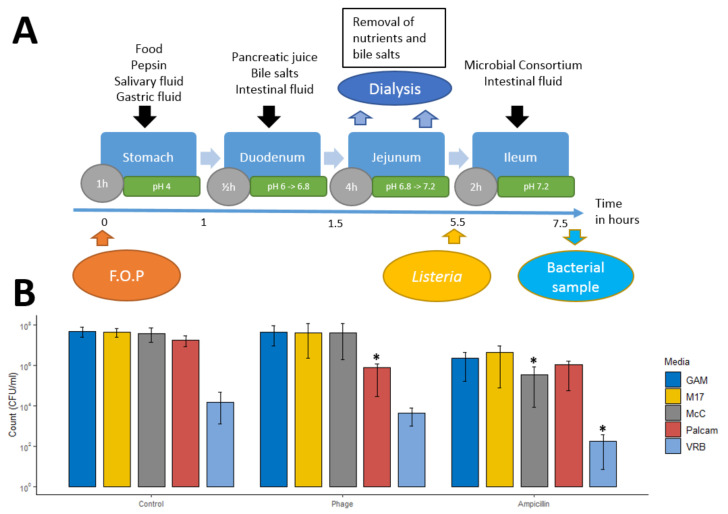
Impact of the FOP bacteriophage cocktail on *L. monocytogenes* in the ileum under simulated small intestinal conditions. (**A**) Overview of experimental setup and sampling. Grey circles denote incubation times for each stage. (**B**) Post ileal concentrations *of L. monocytogenes* and 7 representative bacterial species with FOP and ampicillin treatment (Table 1) [4]. The simulated small intestinal microbiota was enumerated using five different culturing media: Palcam Listeria Selective Agar (Palcam) for enumeration of *L. monocytogenes*, Violet Red Bile Agar (VRB) for enumeration of *E. coli*, M17 Agar (M17) for enumeration of *Streptococcus* sp., MacConkey Agar (MCC) for enumeration of *E. faecalis*, and Gifu Anaerobic Agar (GAM), in which all species from the small intestinal consortium could be cultivated. All experiments were performed in triplicate. Significance was calculated using one-way ANOVA using Tukey’s range test using nontreated samples with *L. monocytogenes* added as controls. * *q* < 0.05.

At the onset of simulated passage of the upper gastrointestinal tract, TSI reactors were inoculated with 0.5 mL of the FOP bacteriophage cocktail (10.81 log PFU/mL, resulting in 9.41 log PFU/mL in the reactor) or ampicillin (500 mg/L in final solution), using saline solution (0.5 mL, 0.9% NaCl) as a control (Figure 1A). Before the ileal step, 1 mL of *L. monocytogenes* strain LM396 suspension (7 log CFU/mL and 1 mL of small intestinal consortium was added into each reactor. Samples were taken from each reactor at the beginning and end of the ileum step, and bacterial enumeration was performed by plate count on Palcam selective agar [19]. The simulated small intestinal microbiota was enumerated using five different culturing media: Palcam Listeria Selective Agar (Palcam selective agar with Palcam selective supplement, Sigma-Aldrich, St. Louis, MO, USA) for enumeration of *L. monocytogenes*, Violet Red Bile Agar (VRB, Sigma Aldrich) for enumeration of *E. coli*, M17 Agar (M17, Oxoid, Basingstoke, UK) for enumeration of *Streptococcus* spp., MacConkey Agar (MCC, Sigma-Aldrich) for enumeration of *E. faecalis* (counting only pink lactic-acid-producing colonies), and Gifu Anaerobic Agar (GAM, Nissui Seiyaku Co., Ltd., Tokyo, Japan), in which all species from the small intestinal consortium could be cultivated. Experiments were conducted for each preparation in triplicate.

Preliminary experiments to test the persistence of the phage cocktail during gastric and small intestinal transit, and its efficacy under simulated intestinal conditions, were performed using only *L. monocytogenes* and the phage cocktail, without adding the small intestinal bacterial consortium. A total of 0.5 mL of FOP was added at the beginning of the stomach stage, and samples were taken at the beginning of the duodenum, jejunum, ileum, and at the end of the ileum stage (Figure 1A). Samples were diluted in SM buffer and refrigerated, and the PFU was determined on the same day.

### 2.5. Colon Model System

#### 2.5.1. Large Intestine Model System

To simulate colonic passage we used the CoMiniGut in vitro colon model [20]. The CoMiniGut consists of five anaerobic reactors with a working volume of 5 mL each. Reactors were filled with basal colon medium mixed with fecal inoculum from an anonymous adult donor (Ethical Committee (E) for the Capital Region of Denmark no. H-20028549) prepared as described previously [20], and parameters such as pH and temperature were monitored and maintained at physiologically relevant levels during simulations. In order to mimic the passage through the colon, the pH was continuously controlled and gradually elevated from pH 5.7 to 6.9 over a 24 h period [20]. Before the start of the experiment, the pH was adjusted to 5.7, and anaerobic conditions were confirmed.

#### 2.5.2. Impact of the FOP Bacteriophage Preparation on *L. monocytogenes* and Colon Microbiome

**Figure 2:**
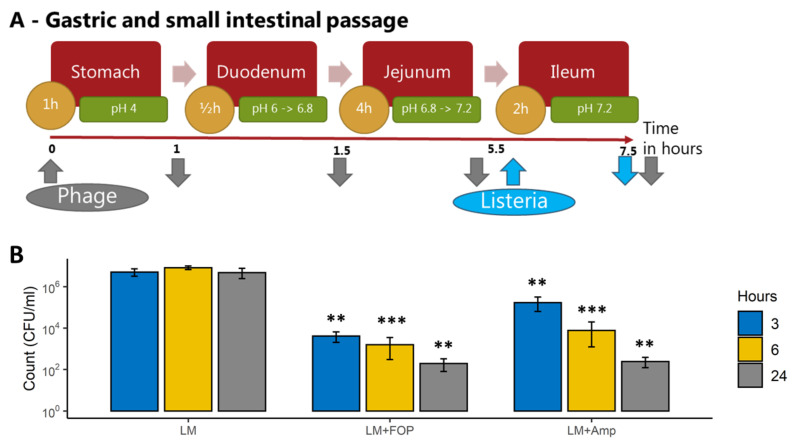
(**A**) Flowchart of the colon (CoMiniGut) simulations showing *L. monocytogenes* (blue) and phage cocktail (grey) additions (upwards arrows) and sampling (downwards arrows) time points. (**B**) Impact of the FOP bacteriophage cocktail and ampicillin treatment on *L. monocytogenes* in the CoMiniGut in vitro colon model. Bacteria and treatments were added to the CoMiniGut reactors followed by sampling at 3, 6, and 24 h. Experiments were performed in triplicate, and *L. monocytogenes* was enumerated by plate count on Palcam Listeria Selective agar. Significance was calculated using one-way ANOVA using Tukey’s range test, using samples with *L. monocytogenes* added without treatment as control values. ** *q* < 0.01, *** *q* < 0.001.

At the start of the experiments, combinations of 0.2 mL of the FOP phage cocktail (9.41 log pFU/mL in the reactors), 10^8^ CFU/mL *L. monocytogenes* strain LM 396, 0.5 ug/mL ampicillin, or 0.9% saline controls were added, and the colon simulation was run as previously described [20]. A total of 200 µL of sample was taken from each reactor at 3, 6, and 24 h (Figure 2A). The number of viable *L. monocytogenes* cells was determined by plate count on PALCAM *Listeria* selective agar. To assess the impact of treatments on overall microbial composition samples, total bacterial DNA was extracted and subjected to 16S rRNA gene amplicon sequencing as described below.

### 2.6. Sequencing of Bacterial Community

#### 2.6.1. Library Preparation and Sequencing

The bacterial community composition was determined by Illumina NextSeq-based high-throughput sequencing (HTS) of the 16S rRNA gene V3-region, according to Krych et al. [21]. Briefly, the amplified fragments with adapters and tags were purified and normalized using custom magnetic carboxylate beads, pooled, and subjected to 150 bp pair-ended Illumina NextSeq (V3 region 16S rRNA) sequencing. The raw dataset containing pair-end reads with corresponding quality scores were merged and trimmed with usearch [22] using the following settings: -fastq_minovlen 100, -fastq_maxee 2.0, -fastq_truncal 4, and -fastq_minlen of 130 bp. Dereplicating, purging from chimeric reads, and constructing de novo zero-radius operational taxonomic units (zOTUs) were conducted using the UNOISE pipeline [23] and taxonomically assigned with sintax [24] coupled to the EZtaxon [25] 16S rRNA gene reference database. Sequences are available at the European Nucleotide Archive (ENA) with accession number PRJEB42055.

#### 2.6.2. Bioinformatic Analysis

Initially, the dataset was purged for zOTUs, which were detected in less than 5% of the samples, but the resulting dataset still maintained 98% of the total reads. R version 4.01 [26] was used for subsequent analysis and presentation of data. Cumulative sum scaling (CSS) [27] was applied for the analysis of beta-diversity to counteract that a few zOTUs represented a majority of count values, since CSS has been benchmarked with a high accuracy for the applied metrics [28]. CSS normalization was performed in R software using the metagenomeSeq package [29]. The alpha-diversity analysis was based on raw read counts, rarified to a median depth of 44,574. The main packages used were phyloseq [30], vegan [31], ggpubr [32], and ggplot2 [33]. Beta-diversity was represented by Bray–Curtis dissimilarity. The data and code used were uploaded as Supplementary Data.

### 2.7. Caco-2 Intestinal Epithelial Model

#### 2.7.1. Caco-2 Cell Culturing

The human colon adenocarcinoma cell line Caco-2 (ATTC HTB-37, LGC standards, Middlesex, UK) at passage 53 was grown in Dulbecco’s Modified Eagle Medium (DMEM) supplemented with 10% (*v*/*v*) heat-inactivated fetal bovine serum (FBS; Lonza, Basel, Switzerland), 1× nonessential amino acids (NEAAs), and 0.1 mg/mL gentamicin. Media were changed 3 times weekly. All solutions were obtained from Invitrogen, Gibco (Naerum, Denmark). The cells were cultured at 37 °C in a humidified atmosphere of 5% CO_2_.

#### 2.7.2. Adhesion and Invasion Assay

Approximately 10^5^ Caco-2 cells per well were seeded in a 24-well microtiter plate in wells coated with 0.1% gelatin and differentiated for 14 days in DMEM supplemented with 20% HI-FBS, 10 mM HEPES, and Antibiotic-Antimycotic solution (Gibco, Thermo Fisher Scientific, Waltham, MA, USA, CAT no. 15240062) at 37 °C in a humidified atmosphere maintained at 5% CO_2_. The medium was changed every 2–3 days. *L. monocytogenes* strain LM 396 was resuspended at 10^8^ CFU/mL in a solution comprising 80% DMEM with 10 mM HEPES and 10% FBS with appropriate amounts of the phage cocktail to achieve a MOI of 10, 100, or 1000. After 30 min of preincubation, the *L. monocytogenes*-phage mixture was added to wells containing the Caco-2 cells and incubated for 1 h. The medium was then removed and saved for plate counting and cytokine measurements andthe Caco-2 cells were washed with Dulbecco’s PBS (DPBS; Sigma-Aldrich, St. Louis, MO, USA). Cells were then either lysed or incubated using DMEM with 10 mM HEPES containing 50 μg/mL gentamicin for 1.5 h. Cells treated with gentamicin were then washed with DPBS and lysed using ice-cold lysis buffer (10 mmol/L Tris pH 7.2, 150 mmol/L NaCl, 1% Triton X-100). For selected dilutions of the lysed cells that did not receive gentamicin, the lysed cells that received the antibiotic, or the medium removed prior to the first wash step (for the wells used for both the adhesion and invasion assay and the invasion assay), 100 μL was spread on LB agar plates and incubated at 37 °C. After 48 h, the number of *L. monocytogenes* colonies was determined. Each trial was performed in triplicate.

#### 2.7.3. Transepithelial Resistance (TER) Assay

The protective effect of FOP on the epithelial barrier exposed to *L. monocytogenes* was evaluated by measurement of TER using the Millicell Electrical Resistance System (Millipore, Bedford, MA, USA) as previously described [34]. To obtain polarized monolayers, Caco-2 cells were seeded onto Transwell filter inserts (0.4 μm pore size, 12 mm inside diameter, polycarbonate; Corning Incorporated, Corning, NY, USA) at a concentration of 2 × 10^5^ cells/mL and cultivated for 14 days, with media change every 2 days. At 90–95% confluence, cells were moved into a cellZcope 2 impedance-based cell-monitoring unit. Treatments were performed after 2–3 days, once TER reached >1800 Ohm/cm^2^. Overnight cultures of bacteria were suspended in cell growth medium without antibiotics. An *L. monocytogenes* LM396 suspension in DMEM was added to the apical compartment at 10^6^ CFU/mL and incubated in a Forma Series 2 Water-Jacketed CO_2_ Incubator (Thermo Fisher, Waltham, MA, USA) at 37 °C in a humidified atmosphere of 5% CO_2_. TER was measured before the addition of the bacteria (time zero) and then at 30 min time intervals, and was expressed as the ratio of TER at time *t* in relation to the initial value (at time zero) for each series. The net value of the TER was corrected for background resistance by subtracting the contribution of cell free filter and the medium (150 Ω). The TER of monolayers without added bacteria represented the control for each experiment. Experiments were performed in triplicate.

#### 2.7.4. Statistics

R version 4.01 [26] was used for statistics and presentation of data (the data and code used were uploaded as Supplementary Materials) using the phyloseq [30], vegan [31], ggpubr [32], and ggplot2 [33] packages. Analysis of variance (ANOVA) and permutational ANOVA (PERMANOVA) were used to evaluate group comparisons using Tukey’s range test and the Bonferroni–Holm method, respectively, for multiple testing correction. Significance was determined at *p* < 0.05 level.

## 3. Results

### 3.1. The FOP Bacteriophage Cocktail Selectively Reduces Listeria monocytogenes in a Small Intestine In Vitro Model

The Smallest Intestine (TSI) model was used to investigate the ability of the bacteriophage cocktail to endure digestive tract conditions and reduce *L. monocytogenes* levels in the ileum. The FOP phage cocktail was added before stomach passage, and *L. monocytogenes* was added at the beginning of the ileum phase of the simulated small intestinal passage (Figure 1A). The pathogen was added directly to the ilium phase to simulate ingestion of the phage cocktail after *L. monocytogenes* had successfully survived stomach passage and infected the lower small intestine. The bacteriophage cocktail caused a significant 1.5-log reduction in *L. monocytogenes* levels (*q* = 0.01) after two hours of ileal passage, while other representative ileal bacteria were not significantly affected (Figure 1B). Ampicillin treatment showed a similar 1.5-log reduction in *L. monocytogenes* (*q* = 0.01), but in contrast to the phage treatment, representative ileal bacteria also showed a 1.5-log reduction on average (Figure 1B). The small intestinal simulation was run using “fed” small intestine conditions (i.e., added food components, stomach pH 4, bile salts = 4 mM, pancreatic juice = 40 U/mL), as stomach pH values of below 3.5 resulted in total phage deactivation (Figure S1). These “fed-state” conditions used adjusted gastric pH values and bile salt concentrations to mimic GI conditions after a meal [16].

### 3.2. The FOP Bacteriophage Cocktail Significantly Reduces L. monocytogenes in a Colon Model While Preserving Bacterial Community Structure

To test the effect of the FOP phage cocktail on *L. monocytogenes* and the overall bacterial community in the colon, we used the CoMiniGut colon in vitro model. Phage cocktail, ampicillin, or saline control was added at the start of the experiment, and the model was run for 24 h to simulate colon passage (Figure 2A). Samples treated with the bacteriophage cocktail had a 3-log reduction (*p* < 0.01) in *L. monocytogenes* CFU at 3 h, and a 5-log reduction (*p* < 0.01) after 24 h of simulated colon passage (Figure 2B). Ampicillin treatment resulted in a similar 2-log reduction of *L. monocytogenes* at 3 h (*p* < 0.01), with a final 5-log reduction (*p* < 0.01) at 24 h, compared to saline-treated control samples.

**Figure 3:**
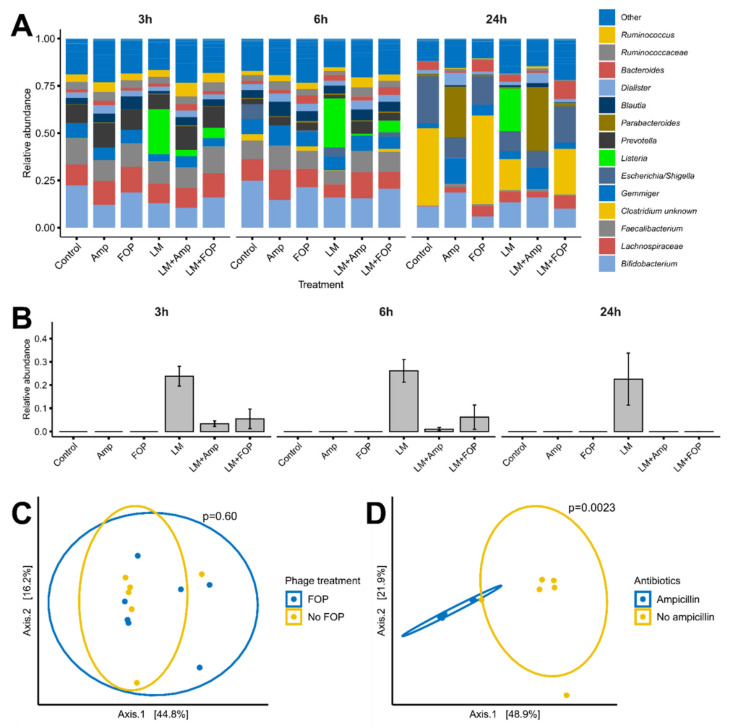
Impact of the FOP bacteriophage cocktail and ampicillin on the bacterial community in the CoMiniGut in vitro colon model, determined by 16S rRNA amplicon sequencing. Bacteria and treatments were added to the CoMiniGut reactors followed by sampling at 3, 6, and 24 h. (**A**) Relative abundance of bacterial genera ordered by average abundance. Legend color order corresponds to chart order. (**B**) Relative abundance of *L. monocytogenes* by treatment over 24 h. (**C**,**D**) PCoA plot of Bray–Curtis dissimilarity metrics after 24 h of in vitro simulated colon passage with FOP or ampicillin treatment. Experiments were performed in triplicate, and significance was calculated using one-way ANOVA using Tukey’s range test.

To determine the overall impact of the phage cocktail on the colon bacterial community structure, 16S rRNA gene amplicon sequencing was performed. While there were shifts in bacterial community composition over time, *L. monocytogenes* was able to persist at a relative abundance of approximately 25% throughout the 24 h of simulated colon passage in nontreated (control) samples (Figure 3A,B). The measured decrease in the relative abundance *L. monocytogenes* showed a similar trend to plate count results, with a decrease to 5% relative abundance at 6 h and below detection limit (0.1%) at 24 h (Figure 3A,B). No significant effects of treatments were seen on alpha diversity, but overall, 24 h samples showed a decrease in alpha diversity (Figure S2). Noteworthy, at 24 h, the bacterial communities treated with the phage cocktail had community structure close to that of the untreated control (Figure 3C), while those treated with ampicillin markedly differed from untreated controls (*p* = 0.002) (Figure 3D and Figure S3). Addition of *L. monocytogenes* had no significant effect on the overall bacterial community composition in nontreated samples at 24 h (*p* = 0.24).

### 3.3. The FOP Bacteriophage Cocktail Significantly Reduces L. monocytogenes Adhesion and Invasion of Caco-2 Cells

**Figure 4:**
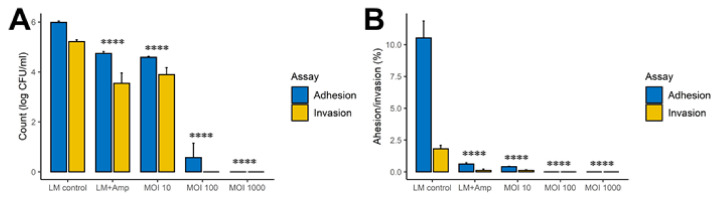
Impact of the FOP bacteriophage cocktail and ampicillin treatment on adhesion and invasion on a Caco-2 cell monolayer. Phage cocktail, ampicillin, or PBS control was added to a *L. monocytogenes* suspension in DMEM and preincubated for 30 min. The preincubated mixtures were added to wells and incubated for one hour. (**A**) Adhesion and invasion CFU counts after 1 h of treatment with phage cocktail (MOI 10–100), ampicillin (500 mg/L), or *L. monocytogenes* alone. (**B**) Percentage adhesion and invasion after 1 h of treatment with phage cocktail, ampicillin (500 mg/L), or *L. monocytogenes* alone. Experiments were performed in triplicate, and *L. monocytogenes* was enumerated by plate count on Palcam *Listeria* Selective agar. Significance was calculated using one-way ANOVA using Tukey’s range test, using samples with *L. monocytogenes* added without treatment as control values. **** *q* < 0.0001.

To examine if the FOP phage preparation had protective effects in the intestine, we used adhesion and invasion assays based on the Caco-2 epithelial cell line. Phage cocktail, ampicillin, or PBS control was added to DMEM media containing *L. monocytogenes* and preincubated for 30 min, added to confluent Caco-2 cell monolayers, and incubated for one hour. The bacteriophage cocktail resulted in a 5-log reduction (*q* < 0.0001) in both adhesion and invasion of *L. monocytogenes*, while ampicillin treatment only resulted in a 1-log reduction (*q* < 0.001) in adhesion and invasion (Figure 4A,B). The reduction was highly dosage dependent, with phage treatment at MOI 10 resulting in 1-log reduction (*p* > 0.0001), while MOI 100 strongly reduced both adhesion and invasion (*p* > 0.0001), and MOI 1000 prevented both adhesion and invasion (*p* > 0.0001).

### 3.4. Cytokine Production and Transepithelial Resistance in Caco-2 Cells

To further assess the protective effect of the phage cocktail on the intestinal epithelium, we measured the cytokine response of Caco-2 cells after one hour of incubation with *L. monocytogenes* with or without 30 min of preincubation with the phage cocktail. However, we did not measure any cytokine response of IFN-γ, IL-1β, IL-6, or TNF-α regardless of treatment, with a detection limit of <0.22 pg/mL (data not shown). Samples treated with the phage cocktail alone also showed no detected effect on cytokine levels.

To measure the ability of the FOP phage cocktail in preserving epithelial integrity, we used a Caco-2 Transwell model to measure the effect on transepithelial resistance (TER) after exposure to *L. monocytogenes* with or without preincubation with the phage cocktail. Initial experiments showed that *L. monocytogenes* treatment led to a rapid drop in TER after 3 h, but phage pretreatment delayed this drop in a dosage-dependent manner (Figure S4A). However, inspection of the wells after the experiment revealed an equally dosage-dependent drop in pH. After the addition of HEPES buffer to stabilize pH, the drop in TER was delayed until after 24 h (Figure S4B). The pH measurements revealed that the pH had decreased again at this time. We therefore concluded that *L. monocytogenes* was able to disrupt the integrity of the Caco-2 monolayer only by lowering the pH of media, and not through direct interaction with the cells. The FOP appeared to have some dosage effect on preserving epithelial integrity, which was likely due to bacteriophages reducing the levels of and/or slowing the growth of the bacteria, delaying the lowering of pH.

## 4. Discussion

In the present study, we demonstrated that the FOP bacteriophage cocktail was able to survive gastric passage and selectively and significantly reduce *L. monocytogenes* in both the ileum and colon under in vitro simulated gastrointestinal conditions.

In the TSI small intestinal in vitro model, treatment with the FOP cocktail led to a significant 1.5-log reduction in the *L. monocytogenes* levels during the relatively short 2 h ileum transit time. In both the ileum and colon systems, the FOP cocktail only reduced *L. monocytogenes*, with no significant impact on any of the other commensal bacteria included in our system. In contrast, ampicillin treatment led to a significant killing-off of commensal small intestinal bacteria, in addition to a reduction in *L. monocytogenes*. In the CoMiniGut in vitro colon model, *L. monocytogenes* CFU counts were significantly reduced following bacteriophage treatment. Treatment by FOP did not alter the composition of the in vitro simulated colon microbiome, demonstrating that FOP retained *L. monocytogenes*-specific bactericidal activity within the complex colonic bacterial community (in addition to *L. monocytogenes*, FOP also targets *Salmonella* spp. and STEC, but representatives of those pathogens were not included in our in vitro system). In the untreated samples, both CFU/mL and relative abundance of *L. monocytogenes* remained relatively stable over the 24 h period. This was in contrast to other studies, which showed that *L. monocytogenes* was able to disrupt the composition of existing microbiota and infect robustly [35], and that certain probiotic intestinal species could inhibit the ability of *L. monocytogenes* to invade and grow [36]. It is possible that we did not observe significant interaction between *L. monocytogenes* and colon bacteria due to the diluted conditions of the TSI and CoMiniGut models relative to the human GI tract. However, the FOP has been shown to not affect the murine microbiome in vivo [14], in correspondence with our results.

Determination of the adhesion and invasion properties of *L. monocytogenes* to a Caco-2 epithelial cell monolayer showed that pretreating *L. monocytogenes* with the FOP had a strong protective effect. This suggested that a sufficient intestinal bacteriophage concentration could prevent *L. monocytogenes* from invading the epithelial barrier. In support of this, a study using a mouse model with oral gavage with a ListShield™ bacteriophage cocktail (the *L. monocytogenes*-targeting component of FOP) was able to reduce the concentration of *L. monocytogenes* in the GI tract, as well as its translocation to the spleen and liver [37]. While high dosage/multiplicity of infection (MOI) appear to be vital for successful bacteriophage treatment [38], production of appropriately high titer preparations is feasible [38,39].

We did not observe any significant effect of *L. monocytogenes* or the phage cocktail on either transepithelial resistance (TER) or cytokine production in the Caco-2 epithelial cell model. Previous studies reported that *L. monocytogenes* strains producing listeriolysin O elicited a persistent IL-6 response [40]. We do not know whether the LM396 strain we used during our studies produced listeriolysin O and/or its expression levels; thus, it is possible that the observed discrepancy between our study and the previous report [40] on the impact of *L. monocytogenes* on IL-6 production was due to LM396 not producing listeriolysin O, or producing it in lower levels compared to the strains used in the previous study. With regard to the FOP phage preparation, our results supported the idea that the phage cocktail itself did not provoke inflammatory response by the epithelial cells, as demonstrated by not eliciting IFN-γ, IL-1β, IL-6, or TNF-α production in the Caco-2 cells.

Phage treatment is most commonly delivered orally, although many other modes of delivery, such as auricular, intravesical, intrapulmonary, rectal, topical, and intravenous, have also been used [41]. Here, we showed that the component of the bacteriophage cocktail targeting *L. monocytogenes* was able to survive gastric conditions, but only when stomach pH was 4 or above. The acidity of the human stomach is highly variable over time and between individuals, but stomach pH values of 4–5 are representative of conditions after meal ingestion [42]. These results agreed with the general observation that phage protein structures are acid labile [43,44]. Furthermore, it is possible that some of the GI conditions not examined in our model systems, such as intestinal peristalsis, complex microbiota, various diets, etc., may further reduce phage viability—and thus efficacy—in vivo. A major condition not included in our model was spatial heterogeneity, since it has been found that sheltered pockets of nonresistant pathogens can survive in vivo in the GI tract simply by spatial separation from the phage-rich main lumen [45]. However, a previous mouse study found that the FOP was able to effectively reduce the concentration of *L. monocytogenes* in the GI tract and its translocation to the spleen and liver when administered 3 days before and after infection [37]. One approach commonly used during therapeutic phage applications in the former Soviet Union and Eastern Europe (and during some animal studies) was to administer oral phage preparations together or shortly after administering sodium bicarbonate to reduce stomach acidity. Alternatively, there is currently a wide range of encapsulation methods available that could be used to formulate lytic phage preparations in “enteric” gel caps or tablets, to ensure phage survival through the stomach and their release in the intestine [46].

## 5. Conclusions

In summary, our data demonstrated that the FOP bacteriophage cocktail could: (i) endure gastric passage under “fed” conditions; and (ii) significantly reduce *L. monocytogenes* in a highly selective manner under in vitro human gastric conditions while having no detectable deleterious effect on the commensal gut microbiota. Furthermore, the data suggested that the phage cocktail had a strong protective effect on adhesion and invasion by *L. monocytogenes* through a Caco-2 monolayer. These results were in agreement with previous reports that FOP could provide robust protection against pathogenic bacteria both in vivo [47] and in vitro [13], while avoiding detrimental effects on the existing microbiota. Taken together, our data provided further support of the idea that lytic phages may provide some important health benefits; e.g., when consumed as dietary supplements, by enhancing natural defenses of the GI tract against specific foodborne bacterial pathogens. For example, phages with strong lytic potency against *L. monocytogenes* included in the FOP preparation may help increase gut resilience against *L. monocytogenes* by specifically killing these bacteria (and preventing their attachment and invasion into epithelial cells) if they are introduced into the gut. Such nutraceutical products (FOP or similar) targeting major foodborne pathogens have the potential to reduce the risk of serious foodborne illnesses caused by consumption of foods that may be contaminated with those pathogens, without eliciting deleterious alterations in the commensal gut microbiota.

## Figures and Tables

**Table 1:** Small intestinal consortium bacterial strains, source, culturing time, and culture media.

Species	Strain Number	Origin	Culture Time (h)	Culture Media
*Escherichia coli*	DSM 1058	Human origin	24	GAM
*Streptococcus salivarius*	DSM 20560	Blood	6	GAM
*Streptococcus luteinensis*	DSM 15350	Human origin	24	GAM
*Enterococcus faecalis*	DSM 20478	Human feces	24	GAM
*Bacteroides fragilis*	DSM 2151	Appendix abscess	24	GAM
*Veillonella parvula*	DSM 2008	Human intestine	48	GAM
*Flavonifractor plautii*	DSM 6740	Human feces	48	GAM

GAM = Gifu Anaerobic Medium. Modified from Cieplak et al. 2018 [16].

## Data Availability

Sequences are available at the European Nucleotide Archive (ENA) under accession number PRJEB42055 (https://www.ebi.ac.uk/ena/browser/view/PRJEB42055).

## Supplementary Materials

The following are available online at https://www.mdpi.com/article/10.3390/v14020190/s1, Table S1. Current bacteriophage composition of the FOP phage preparation, Figure S1. Phage survival by stomach step pH, Figure S2. Bacterial alpha diversity measured by observed OTUs and Shannon diversity index, based on 16S rRNA sequencing, Figure S3. PCoA plot of Bray–Curtis dissimilarity distance for all time points and treatments, Figure S4. (A) Dosage effect of *L. monocytogenes* on transepithelial resistance (TER). (B) Effect of bacteriophage pre-treatment on trans-epithelial resistance (TER) after addition of HEPES buffer to stabilize pH.
