# Supplementary material for: A Bacteriophage Cocktail Significantly Reduces Listeria monocytogenes without Deleterious Impact on the Commensal Gut Microbiota under Simulated Gastrointestinal Conditions
Source: Viruses. 2022 Jan 19;14(2):190. doi: 10.3390/v14020190 (PMC8875722; doi:10.3390/v14020190)
Supplement: Supplementary file 1 [file viruses-14-00190-s001.zip › viruses-1520241-supplementary.pdf]

viruses

Supplementary materials

Table S1. Current bacteriophage composition of the FOP phage preparation.

Species	Strain Source	Origin	Culture Time [h]	Culture Media
Escherichia coli	DSM 1058	Human origin	24	GAM
Streptococcus salivarius	DSM 20560	Blood	6	GAM
Streptococcus luteinensis	DSM 15350	Human origin	24	GAM
Enterococcus faecalis	DSM 20478	Human faeces	24	GAM
Bacteroides fragilis	DSM 2151	Appendix abscess	24	GAM
Veillonella parvula	DSM 2008	Human intestine	48	GAM
Flavonifractor plautii	DSM 6740	Human faeces	48	GAM

GAM = Gifu Anaerobic Medium. Modified from Cieplak et al. 2018.

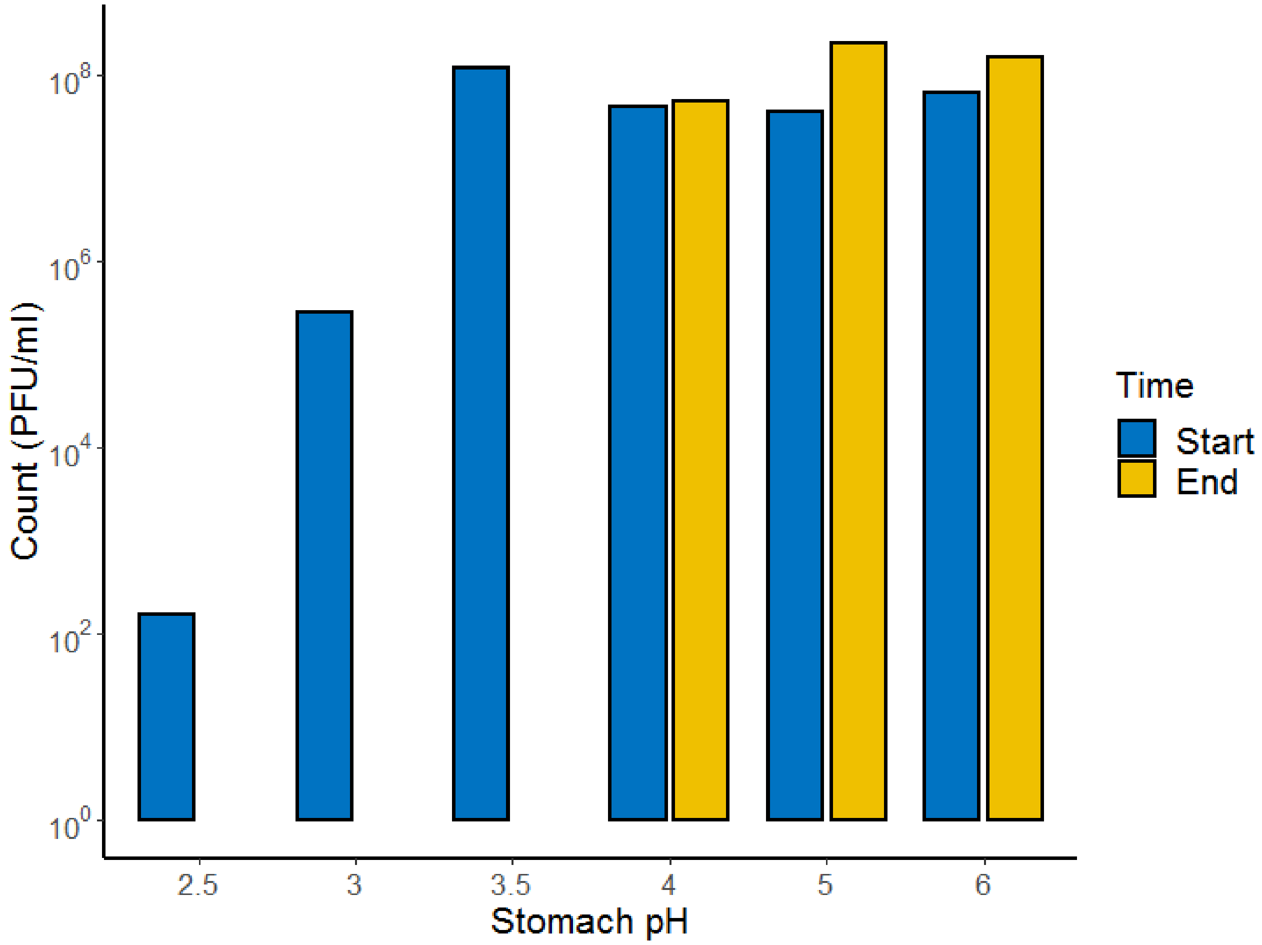

Figure S1. Phage survival by stomach step pH. Samples were collected at the beginning and of the stomach step and at the end of the ileum phase of the TSI simulation and plaque forming units were measured by plaque assay. All TSI simulations were run using standard parameters, apart from presence/absence of feed and pH adjustment. pH values of < 3 were performed without feed to sim-ulated fasted conditions. Runs were performed in duplicate.

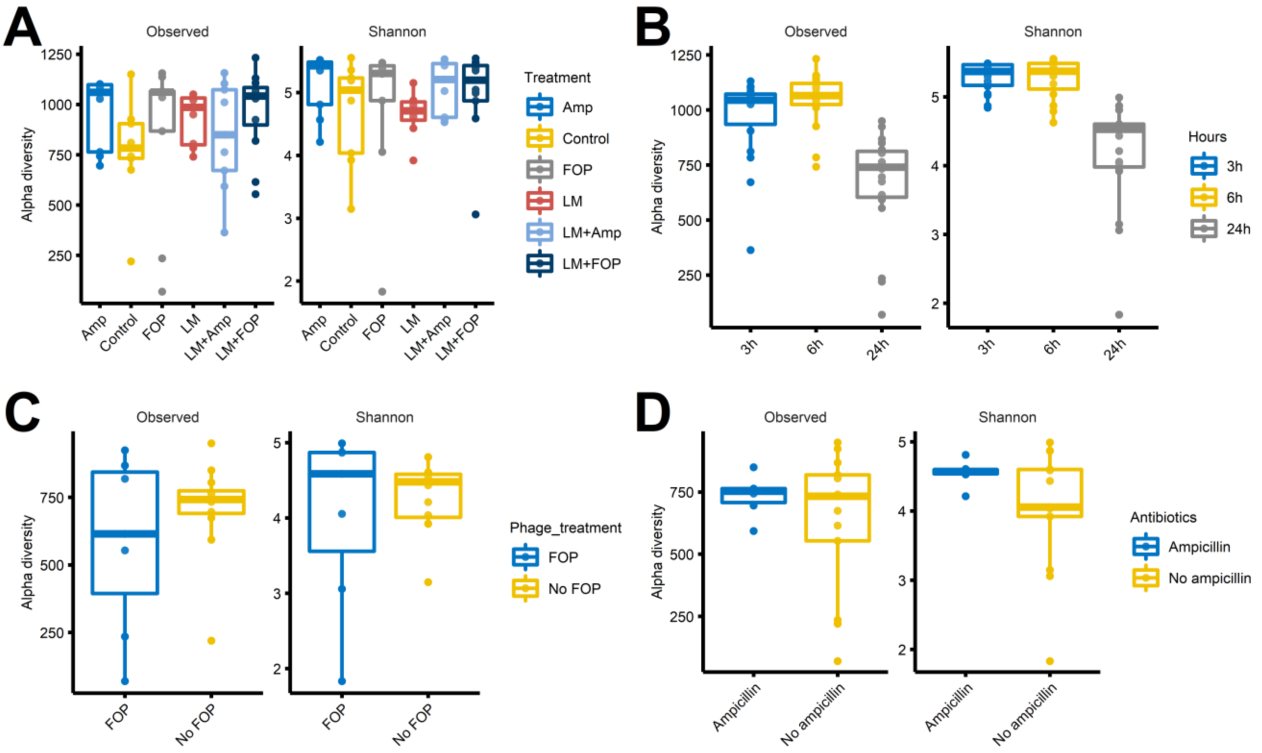

Figure S2. Bacterial alpha diversity measured by observed OTUs and Shannon diversity index, based on 16S rRNA sequencing. (**A**) Alpha diversity by treatment at 24 h. No significant difference. (**B**) Alpha diversity by time points. Both observed zOTUs and Shannon diversity at was significantly lower at 24 h (q < 0.0001). (**C**) Alpha diversity by phage treatment at 24 h. No significant difference. (**D**) Alpha diversity by ampicillin treatment at 24 h. No significant difference. All significances were calculated using ANOVA with using Tukey's range test.

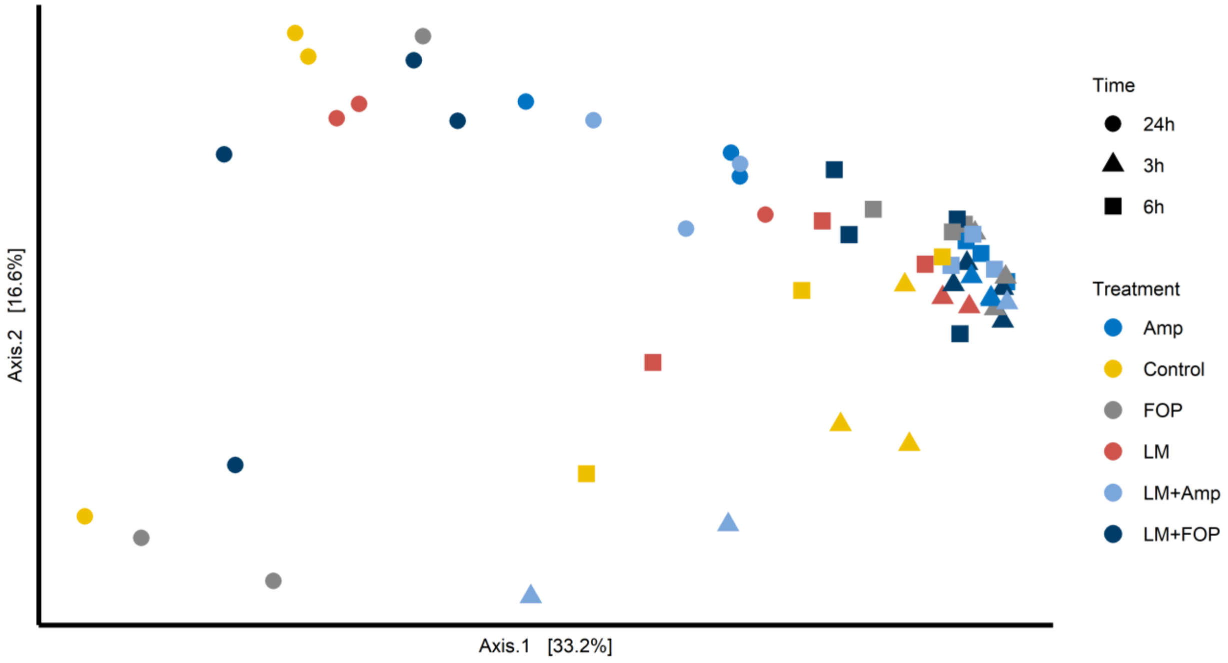

Figure S3. PCoA plot of Bray-Curtis dissimilarity distance for all time points and treatments. Bacte-ria and treatments were added to the CoMiniGut reactors followed by sampling at 3, 6 and 24 h, community composition was determined by 16S rRNA amplicon sequencing.

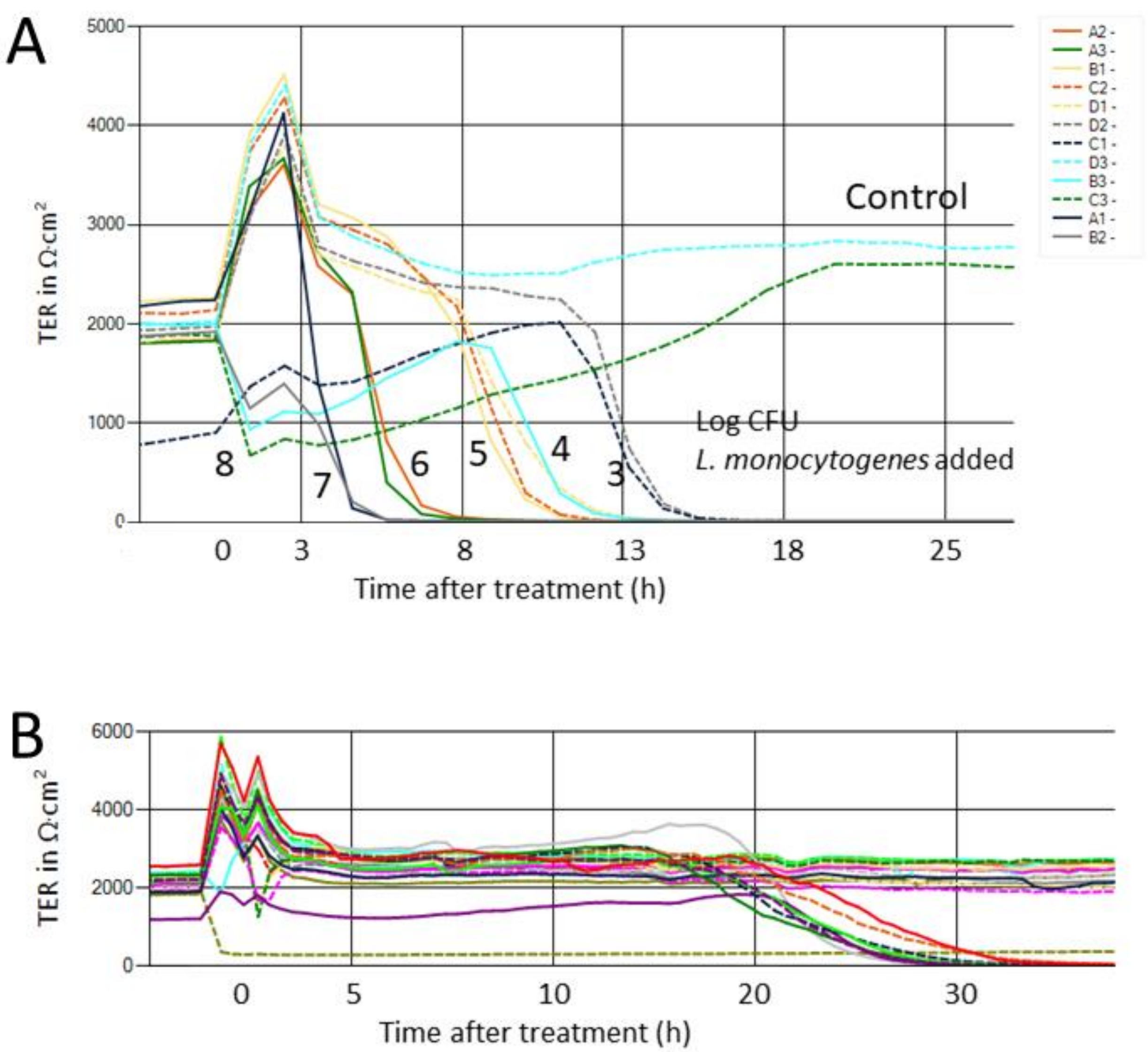

Figure S4. (**A**) Dosage effect of *L. monocytogenes* on trans-epithelial resistance (TER). Each set of colored lines represent a duplicate experiment with different concentrations of bacteria added. Numbers on graph denote log CFU *L. monocytogenes* added. Duplicate experiments were performed for each dose. (**B**) Effect of bacteriophage pre-treatment on trans-epithelial resistance (TER) after addition of HEPES buffer to stabilize pH.
